# Seroprevalence and optimized quantitative PCR detection of *Entamoeba histolytica* using self-collected rectal swabs among men who have sex with men in southern Taiwan: a cross-sectional study

**DOI:** 10.1186/s41182-026-00919-5

**Published:** 2026-02-18

**Authors:** Chin-Shiang Tsai, Koji Watanabe, Akira Kawashima, Wei-Chen Lin

**Affiliations:** 1 Department of Internal Medicine, National Cheng Kung University Hospital, College of Medicine, National Cheng Kung University, Tainan, Taiwan; 2 Department of Parasitology, Division of Host Defense Mechanism, Tokai University School of Medicine, Isehara, Japan; 3 AIDS Clinical Center, National Center for Global Health and Medicine, Japan Institute for Health Security, Tokyo, Japan; 4 Department of Parasitology, College of Medicine, National Cheng Kung University, Tainan, Taiwan

**Keywords:** *Entamoeba histolytica*, Amebiasis, Men who have sex with men, Quantitative PCR, Self-collected rectal swabs, Seroprevalence, Taiwan, Sexually transmitted enteric infections

## Abstract

**Background:**

Amebiasis, caused by *Entamoeba histolytica*, has emerged as a sexually transmitted enteric infection among men who have sex with men (MSM) and people with HIV (PWH) in developed countries. While previous surveillance studies in Taiwan among PWH showed ongoing transmission, investigation on *E. histolytica* infection among non-HIV MSM using newer diagnostic methods and ELISA-IgG seroprevalence surveys is lacking.

**Methods:**

From March 2024 to February 2025, we conducted a cross-sectional study during an LGBTQ + Pride event and at a tertiary medical center in southern Taiwan. MSM participants were recruited during anonymous HIV and syphilis testing, or during follow-up visits for HIV pre-exposure prophylaxis or antiretroviral therapy. We collected serum samples for *E. histolytica* ELISA IgG and self-collected rectal swabs for molecular diagnosis. DNA extraction was performed using an optimized protocol. A novel quantitative PCR assay utilizing specific primers and a double-quencher probe was developed to enhance detection specificity and reduce background noise.

**Results:**

Among 139 non-HIV MSM participants, 11 individuals tested positive for *E. histolytica* IgG, yielding a seroprevalence of 7.9%. Molecular testing by rectal swab quantitative PCR identified two positive cases (1.4%). Of the two positive cases, one developed diarrhea just before examination, while the other remained asymptomatic. The novel PCR primer probe set detected *E. histolytica* DNA from rectal swab samples with high specificity.

**Conclusions:**

The present study investigated *E. histolytica* infection among non-HIV MSM in Taiwan using ELISA IgG and novel molecular diagnostic methods. The seroprevalence of 7.9% suggests ongoing transmission in this population. The optimized self-collected rectal swab protocol combined with the novel PCR assay provides a practical surveillance tool. Further investigation and targeted prevention strategies are warranted.

**Supplementary Information:**

The online version contains supplementary material available at 10.1186/s41182-026-00919-5.

## Background

*Entamoeba histolytica*, the causative agent of amebiasis, is increasingly recognized as a sexually transmitted enteric infection among men who have sex with men (MSM) and people with HIV (PWH) in developed countries [1]. The infection can manifest as asymptomatic luminal colonization, amoebic colitis, or invasive extraintestinal disease, with transmission occurring through fecal–oral contact during sexual activities [2].

Previous studies in Taiwan utilized indirect hemagglutination assay (IHA) to investigate seroprevalence and in-house PCR to confirm luminal amebiasis among PWH, demonstrating ongoing transmission in this population [3, 4]. However, comprehensive investigation on *E. histolytica* infection among non-HIV MSM using modern diagnostic methods remains limited. Furthermore, rectal screening using self-collected specimens and seroprevalence surveys employing standardized ELISA-IgG assays have not been conducted in Taiwan.

Accurate diagnosis of *E. histolytica* infection requires differentiation from the morphologically identical but non-pathogenic *Entamoeba dispar*. Molecular diagnostic methods, particularly quantitative PCR (qPCR), offer superior specificity and sensitivity compared to microscopy [5]. Recent advances in probe design, including double-quencher probes, have further enhanced detection specificity by reducing background fluorescence and minimizing false-positive results [6].

Self-collected rectal swabs have emerged as a practical, non-invasive sampling method for detecting rectal gonorrhea or chlamydia in MSM populations [7], but evaluation of self-collected rectal swabs for detection of amebiasis is lacking [8]. This approach is particularly valuable for surveillance studies and screening programs targeting high-risk populations.

The objectives of this study were to: (1) determine the seroprevalence of *E. histolytica* infection among non-HIV MSM in southern Taiwan using ELISA-IgG; (2) evaluate the utility of self-collected rectal swabs combined with an optimized qPCR assay for molecular detection; and (3) assess the feasibility of this surveillance approach for identifying asymptomatic carriers in high-risk populations. Analyses among people with HIV (PWH) were exploratory and descriptive, intended to provide contextual comparison rather than formal subgroup inference.

## Materials and methods

### Study design and setting

This cross-sectional study was conducted from March 2024 to February 2025 at two sites in southern Taiwan: an LGBTQ + Pride event held in March 2024 and the outpatient clinics of National Cheng Kung University Hospital, a 1300-bed tertiary medical center in southern Taiwan. The study protocol was approved by the Institutional Review Board of National Cheng Kung University Hospital (approval number A-BR-113-009).

### Study participants

MSM status was self-reported based on sexual behavior, defined as having had sexual contact with men. MSM participants aged 18 years or older were recruited through two pathways: (1) during anonymous HIV and syphilis testing at the LGBTQ + Pride event, and (2) during routine follow-up visits for HIV pre-exposure prophylaxis (PrEP) or HIV antiretroviral therapy (ART) at the hospital. All participants provided written informed consent. All testing was provided free of charge, and no incentives or reimbursements were offered. Exclusion criteria included inability to provide informed consent or refusal to provide biological specimens.

### Specimen collection

After obtaining informed consent, participants provided: (1) serum samples (5 mL) for *E. histolytica* ELISA IgG testing, and (2) self-collected rectal swabs for molecular diagnosis. For rectal swab collection, participants were instructed to insert the FecalSwab^™^ (Copan Diagnostics, USA) containing Cary–Blair transport medium approximately 3–5 cm into the rectum, rotate gently, and place it back into the tube. Specimens were collected immediately after defecation when possible. Swabs were processed within 72 h of collection and stored at −80 °C until DNA extraction. Gastrointestinal symptoms were collected using a brief standardized questionnaire at the time of specimen collection. Symptom assessment was included in the IRB-approved protocol and informed consent materials.

### Serological testing

Serum sampling and self-collected rectal swab collection were performed on the same day for the majority of participants. Serum samples were tested for *E. histolytica*-specific IgG antibodies using a commercial ELISA kit (NovaTec Immundiagnostica GmbH, Dietzenbach, Germany) according to the manufacturer’s instructions. Results were interpreted as positive, negative, or equivocal based on the optical density values relative to the cutoff calibrator.

### DNA extraction from rectal swabs

**Fig. 1:**
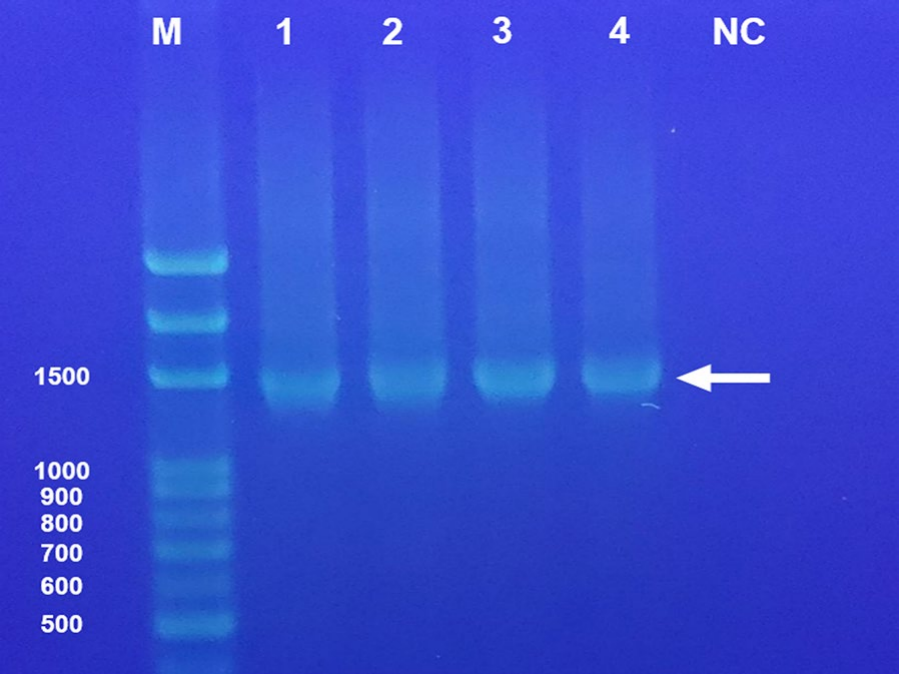
Detection of the prokaryotic 16S rRNA gene by agarose gel electrophoresis, showing an approximately 1500 bp long (arrow), suggesting DNA extraction from rectal swab samples. Lane M, 100 bp DNA marker, lanes 1 and 2 represent positive cases, while lanes 3 and 4 are negative cases in the following *E. histolytica* qPCR. *NC* negative control

DNA extraction protocol (Table 1) consisted of the following steps: (1) rectal swabs were vortexed for 1 min to release material into the Cary–Blair medium; (2) the preservation solution (approximately 1.7 mL) was transferred to a 2.0 mL microcentrifuge tube; (3) Tween-20 was added to achieve a final concentration of 0.02–0.05% and vortexed for 30–60 s; (4) samples were centrifuged at 10,000 g for 3 min; (5) the supernatant was carefully removed, leaving 200 μL; and (6) the resulting pellet was immediately processed using the QIAamp Fast DNA Stool Mini Kit (Qiagen, Hilden, Germany) following the manufacturer's instructions, or stored at −80 °C for up to 1 month until extraction. DNA quality was verified by spectrophotometry (NanoDrop, Thermo Fisher Scientific, USA) and bacterial DNA extraction was confirmed by amplification of the prokaryotic 16S rRNA gene, yielding an approximately 1,500-bp product visualized by agarose gel electrophoresis (Fig. 1).

**Table 1:** Optimized protocol for DNA extraction from rectal swab samples

Step	Protocol details
Sample collection	Use Copan FecalSwab with Cary–Blair transport medium to collect as much stool as possible. Collection should be performed immediately after defecation. After sampling, insert swab top into the green-capped tube, fold at red line, and close cap tightly
Sample processing	Vortex well (about 1 min) before opening the lid. Open lid, discard swab, and transfer preservation solution (approximately 1.7 mL) to a 2.0 mL centrifuge tube. Add Tween solution to achieve 0.02–0.05% final concentration and vortex for 30–60 s
Centrifugation	Centrifuge at 10,000 g for 3 min. Remove supernatant carefully, leaving 200 μL. The resulting precipitation contains the bacterial cells for DNA extraction
Storage	Use all precipitation immediately for DNA extraction or store in deep freezer for up to 1 month until extraction is performed. Process preservation solution within 72 h of initial sampling

### Quantitative PCR assay

A novel qPCR assay was developed utilizing specific primers and a double-quencher probe targeting *E. histolytica*-specific sequences. The primer sequences were: forward primer 5'-GCGGACGGCTCATTATAACA-3', reverse primer 5'-CATTGGTTACTTGTTAAACACTGTGTG-3'. The probe sequence was 5'-/56-FAM/TC ATT + GAA T/ZEN/ + G AAT T + G + G CCA TTT/3IABkFQ/-3', featuring a double-quencher design with an internal ZEN quencher and Iowa Black FQ (3IABkFQ) terminal quencher to minimize background fluorescence. Each qPCR reaction (20 μL) contained 10 μL TaqMan Fast Advanced Master Mix 2 × buffer (Thermo Fisher Scientific, USA), 5 pmol of each primer, 5 pmol of probe, and 2 μL of DNA template. Thermal cycling conditions consisted of initial denaturation at 95 °C for 30 s, followed by 50 cycles of 95 °C for 5 s and annealing at 62 °C for 30 s. Each sample was tested in duplicate. Cycle threshold (Ct) values ≥ 36 were considered negative. The Ct threshold (≥ 36) was defined based on assay validation experiments, including serial dilution testing, replicate concordance, and absence of amplification in negative controls. The diagnostic performance of this primer–probe set was validated by digital droplet PCR (ddPCR) at the AIDS Clinical Center, National Center for Global Health and Medicine, Japan Institute for Health Security, Tokyo, Japan [7]. Positive control samples were derived from the reference strain HM-1:IMSS (obtained from the National Institute of Infectious Diseases, Japan Institute for Health Security, Japan) with an initial concentration of 10^5^ trophozoites/μL. Serial tenfold dilutions were performed to establish assay sensitivity, demonstrating detection down to 10^2^ trophozoites/μL.

### Statistical analysis

Seroprevalence and molecular detection rates were calculated as proportions with 95% confidence intervals (CI). Descriptive statistics were used to summarize participant characteristics and clinical findings. All analyses were performed using R software version 4.3.0 (R Foundation for Statistical Computing, Vienna, Austria).

## Results

**Fig. 2:**
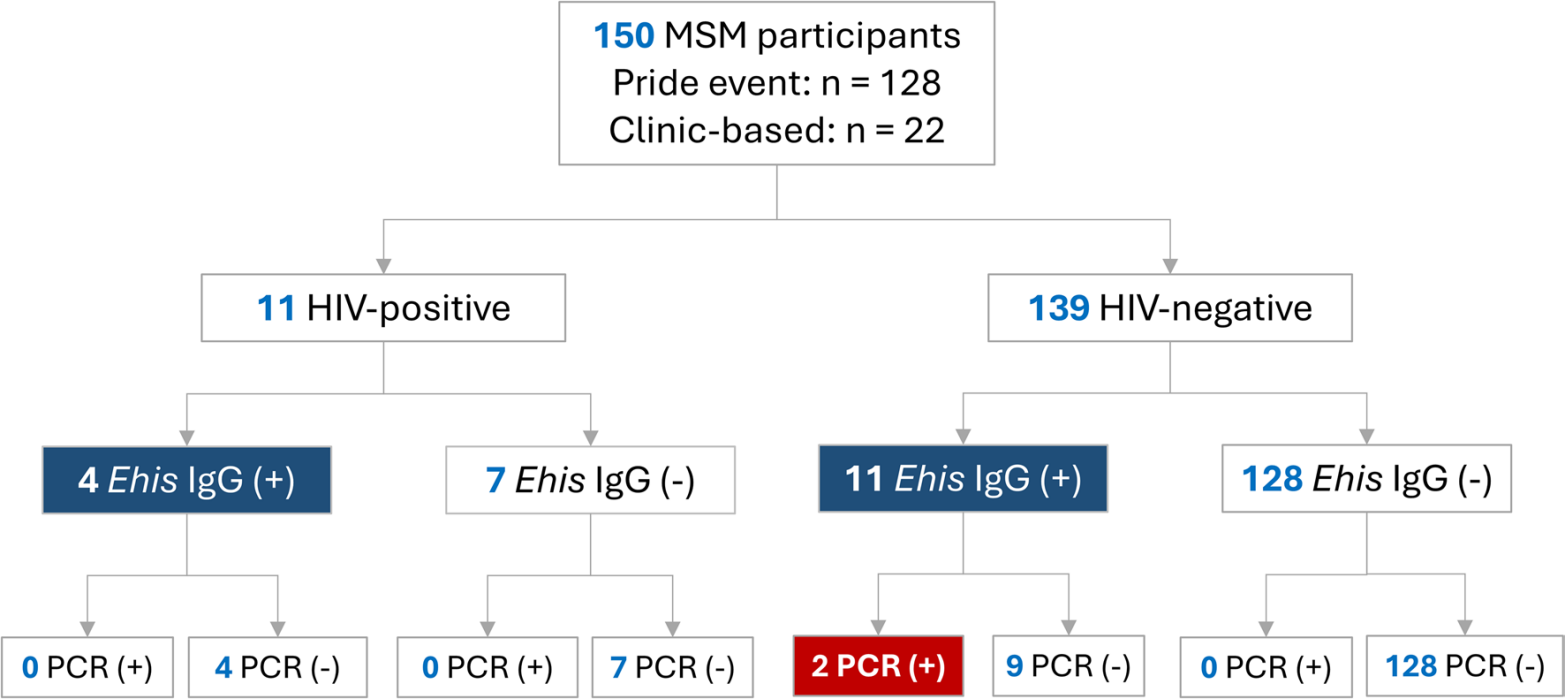
Study flowchart showing participant recruitment and *E. histolytica* testing results. *Ehis Entamoeba histolytica*, *MSM* men who have sex with men, *PCR* polymerase chain reaction, *PWH* people with HIV

A total of 150 MSM participants were enrolled, including 139 non-HIV MSM and 11 PWH. Most participants (128/150, 85.3%) were recruited during an LGBTQ + Pride event, with the remainder recruited during clinic-based follow-up (Fig. 2). Because recruitment pathways may influence underlying prevalence, *E. histolytica* IgG seropositivity and rectal-swab qPCR positivity were further summarized by recruitment pathway and HIV status (Table S1).

Among non-HIV MSM, the seroprevalence of *E. histolytica* IgG was 7.9% (11/139; 95% CI 4.0–13.6%). Rectal-swab qPCR identified two positive cases, yielding a molecular detection rate of 1.4% (2/139; 95% CI 0.2–5.1%). Of these, one individual reported acute diarrhea at the time of sampling, whereas the other was asymptomatic, consistent with luminal colonization.

Among PWH, 4 of 11 participants (36.4%; 95% CI 10.9–69.2%) were seropositive for *E. histolytica* IgG; however, no rectal-swab samples from this subgroup were qPCR-positive.

**Fig. 3:**
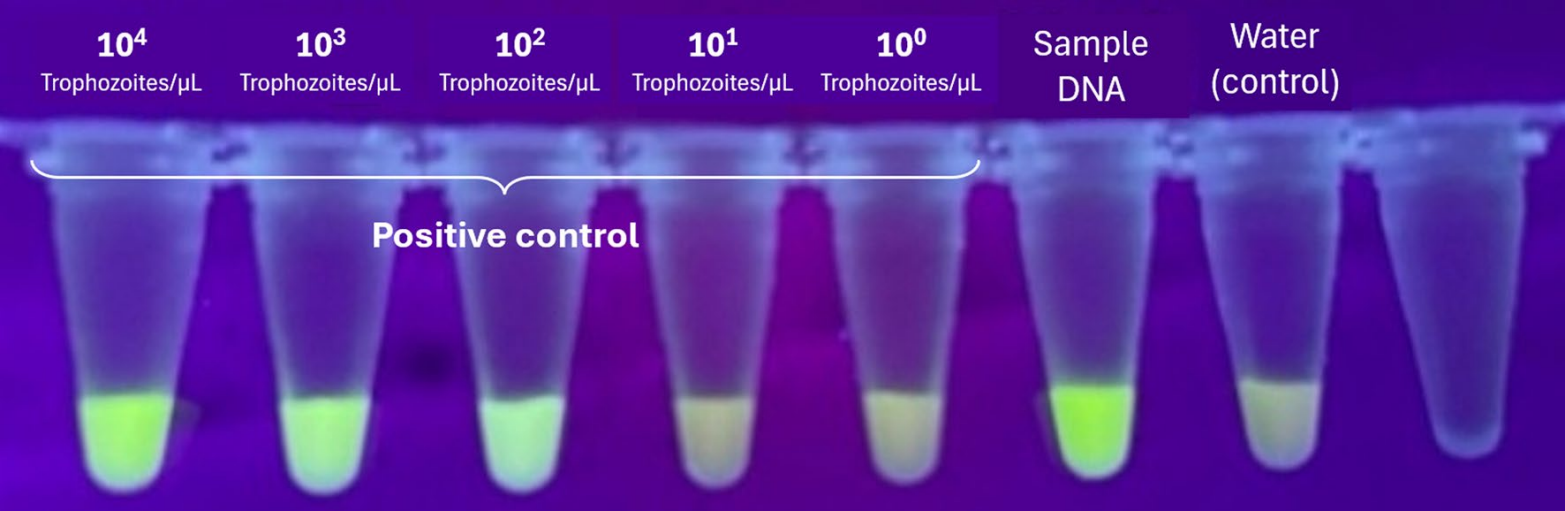
Quantitative PCR amplification curves demonstrating assay sensitivity and specificity. Real-time PCR amplification curves showing detection of *E. histolytica* DNA. Positive control samples were derived from reference strain HM-1:IMSS at an initial concentration of 10^5^ trophozoites/μL, with tenfold serial dilutions. Amplification was observed at dilutions of 10^4^, 10^3^, and 10^2^ trophozoites/μL, yielding Ct values of 23.95, 31.47, and 35.08, respectively. DNA from a confirmed positive clinical case showed Ct value of 27.46. Dilutions of 10^1^ and 10^0^ trophozoites/µL showed no amplification. Water negative control showed no amplification, confirming assay specificity. Ct values ≥ 36 were considered negative

The optimized DNA extraction protocol yielded high-quality DNA from all 150 rectal-swab samples (100%), as confirmed by spectrophotometry and successful 16S rRNA gene amplification. No extraction failures or PCR inhibition events were observed. qPCR was performed for all samples, with E. histolytica DNA detected only among non-HIV MSM. The optimized qPCR assay detected *E. histolytica* DNA at concentrations as low as 10^2^ trophozoites/μL in serial dilution experiments using positive control samples (10^5^ trophozoites/μL), as shown in Fig. 3. Ct values for the tenfold dilution series yielded: 10^4^ trophozoites/µL (Ct 23.95), 10^3^ trophozoites/µL (Ct 31.47), and 10^2^ trophozoites/µL (Ct 35.08). No amplification was observed in negative controls (DNA-free water).

## Discussion

This proof-of-concept study investigates *E. histolytica* infection among non-HIV MSM in Taiwan using both serological and qPCR-based diagnostic approaches. The molecular detection rate of 1.4% by rectal swab qPCR provides evidence of active luminal infection, including both symptomatic and asymptomatic cases. The observed seroprevalence of 7.9% in non-HIV MSM was lower than PWH [4], but higher than a prior study in Taiwan, with 1.42% of MSM seeking voluntary counseling and testing being seropositive [9]. Studies from Japan reported seroprevalence rates of 6.7% among HIV-negative MSM [10]. The detection of one asymptomatic carrier highlights the importance of molecular screening in high-risk populations, as asymptomatic individuals can serve as reservoirs for continued transmission [1]. Self-collected rectal swabs combined with our optimized qPCR assay offer a practical, acceptable, and sensitive surveillance tool for identifying both symptomatic and asymptomatic infections. This approach addresses key barriers to screening, including patient discomfort with clinician-collected specimens and the need for frequent testing in high-risk populations [11].

The novel qPCR assay employing a double-quencher probe design demonstrated satisfying analytical performance, successfully detecting *E. histolytica* DNA at low parasite densities. A prior study showed the double-quencher configuration, utilizing both an internal ZEN quencher and a terminal Iowa Black FQ quencher, effectively reduces background fluorescence and minimizes false-positive results [6]. This is particularly valuable when testing specimens from asymptomatic individuals, where parasite loads may be low.

Several limitations should be acknowledged. First, this study was not designed as a diagnostic accuracy study comparing rectal swab PCR with stool microscopy or stool PCR, which are currently considered reference methods for intestinal amebiasis. Rectal swab PCR may therefore underestimate true prevalence. While we demonstrated the feasibility of detecting *E. histolytica* DNA from self-collected rectal swabs, representing the first report in the literature, further study is needed to validate the performance of rectal swab PCR. Second, the study was conducted at a single geographic location and may not be generalizable to other regions of Taiwan or other countries. Recruitment at an LGBTQ+ Pride event may have introduced selection bias, potentially limiting generalizability to other MSM settings. Third, the small number of participants with HIV limits the precision of descriptive comparisons by HIV status. Fourth, we did not collect detailed sexual behavioral data, preventing analysis of risk factors associated with *E. histolytica* infection. Fifth, the cross-sectional design does not allow assessment of infection dynamics or transmission patterns over time. ELISA IgG reflects prior exposure rather than current infection, and asymptomatic carriers may be seronegative [12].

Despite these limitations, our findings demonstrate the successful implementation of self-collected rectal swabs combined with an optimized qPCR assay, providing a practical surveillance tool for detecting both symptomatic and asymptomatic infections. Our findings highlight the need for enhanced awareness among healthcare providers, routine screening programs in high-risk populations, and targeted prevention strategies emphasizing safe sexual practices. Future longitudinal studies with larger sample sizes are warranted to characterize transmission dynamics, identify risk factors, and evaluate the effectiveness of prevention interventions.

## Supplementary Information

Supplementary material 1. Table S1. Stratified prevalence of *Entamoeba histolytica *IgG seropositivity and rectal-swab qPCR positivity by recruitment pathway and HIV status.

## Data Availability

All data generated or analyzed during this study are included in this published article.

## Abbreviations

MSM: Men who have sex with men
PWH: People with HIV
HIV: Human immunodeficiency virus
PrEP: Pre-exposure prophylaxis
ART: Antiretroviral therapy
ELISA: Enzyme-linked immunosorbent assay
IgG: Immunoglobulin G
IHA: Indirect hemagglutination assay
PCR: Polymerase chain reaction
qPCR: Quantitative polymerase chain reaction
ddPCR: Digital droplet PCR
Ct: Cycle threshold
NIID: National Institute of Infectious Diseases
CI: Confidence intervals
