# Supplementary material for: Seroprevalence and optimized quantitative PCR detection of Entamoeba histolytica using self-collected rectal swabs among men who have sex with men in southern Taiwan: a cross-sectional study
Source: Trop Med Health. 2026 Feb 18;54:30. doi: 10.1186/s41182-026-00919-5 (PMC12922407; doi:10.1186/s41182-026-00919-5)
Supplement: Supplementary file 1 — Supplementary material 1. Table S1. Stratified prevalence of Entamoeba histolytica IgG seropositivity and rectal-swab qPCR positivity by recruitment pathway and HIV status. [file 41182_2026_919_MOESM1_ESM.docx]

**Supplementary Table S1. Stratified prevalence of *Entamoeba histolytica* IgG seropositivity and rectal-swab qPCR positivity by recruitment pathway and HIV status**

| Pathway | HIV status | No. of participants | *E. histolytica* IgG seropositive, n (%) | Rectal swab qPCR positive, n (%) |
| --- | --- | --- | --- | --- |
| Pride event | Non-HIV | 128 | 8 (6.3) | 1 (0.8) |
| Pride event | PWH | — | — | — |
| Clinic follow-up | Non-HIV | 11 | 3 (27.3) | 1 (9.1) |
| Clinic follow-up | PWH | 11 | 4 (36.4) | 0 (0) |

Abbreviations: PWH, people with HIV. No participants with HIV were recruited through the Pride event. Data are presented descriptively to facilitate interpretation of prevalence estimates by recruitment pathway and HIV status. No statistical comparisons were made.
